# The impact factor of journals should not be so valued

**DOI:** 10.5935/0004-2749.2024-1011

**Published:** 2024-08-30

**Authors:** Newton Kara Junior

**Affiliations:** 1 Ophthalmology Department, Universidade de São Paulo, São Paulo, SP, Brazil; 2 Editor-in-Chief of Arquivos Brasileiros de Oftalmologia

Researchers are individuals who are not satisfied with existing explanations and who delve into the scientific method to confirm new theories. Thus, the researcher has an unsatisfied curiosity that motivates the advancement of knowledge. In recent decades, knowledge has evolved faster due to the ease of dissemination via the internet, and information should not only be produced but also be well-disseminated.

Due to the credibility of the editorial process and easy access through bibliographic databases, journals have become vehicles for disseminating science. Therefore, the researcher’s priority after completing a study is to publish in a scientific journal to share their findings with scholars worldwide.

By analyzing the context of the production and dissemination of scientific knowledge in Brazil, we realized that many research groups are linked to *stricto sensu* postgraduate programs; and the main stimulus to publish articles in high-impact factor (IF) journals is because of the pressure from *Coordenação de Aperfeiçoamento de Pessoal de Nível Superior* CAPES (a government body evaluating and promoting postgraduate programs) in evaluating the professor and the program.

The government’s objective, when financing science, is to stimulate good-quality research to improve the nation’s technological performance. In addition to research grants, it relies on CAPES, which directs postgraduate programs to enhance publications in good IF journals and innovations/patents^(1)^.

However, why is the journal’s IF so important? It should not be important because other researchers will certainly read and cite good-quality research published in a journal indexed in the bibliographic databases. Thus, it gives more credibility to cite studies published in famous journals, but a well-designed and relevant study will influence and be cited by other authors, regardless of where it was published. This is because keywords are more important than the name of the journal in searching the literature.

In Brazil, the IF of journals is used as a reference for academic progress, selection of funding grants, and evaluation of postgraduate programs; that is, factors peripheral to the essence of the research objective, which is to contribute to increasing scientific knowledge. Additionally, for adequate knowledge dissemination in the research, the article should be published in any journal indexed in the main bibliographic databases, regardless of the journal’s IF.

We suggest that CAPES should measure the quality of articles by the number of citations, instead of the IF of the publishing journal, which does not necessarily reflect the quality of all articles published there. Because of the need to publish in these journals, researchers pay high publication fees, which do not guarantee that their study will be made available in open access. Thus, excessively valuing IF makes science more expensive and less accessible.

Currently, CAPES has been consistent with the country’s needs, subsidizing publications in national periodicals and sponsoring journals that do not charge authors. However, we believe that CAPES could further support periodicals indexed in the main bibliographic databases and that authors or readers should not be charged (called diamonds), and the journal’s IF should not be prioritized so much.
